# Antifungal Activity of Selected Indigenous *Pseudomonas* and *Bacillus* from the Soybean Rhizosphere

**DOI:** 10.1155/2009/572049

**Published:** 2009-11-25

**Authors:** M. León, P. M. Yaryura, M. S. Montecchia, A. I. Hernández, O. S. Correa, N. L. Pucheu, N. L. Kerber, A. F. García

**Affiliations:** ^1^Instituto de Investigaciones en Biociencias Agrícolas y Ambientales (INBA-CONICET), Facultad de Agronomía, Universidad de Buenos Aires, Av. San Martín 4453, C1417DSE, Ciudad Autónoma de Buenos Aires, Argentina; ^2^Cátedra de Microbiología Agrícola, Facultad de Agronomía, Universidad de Buenos Aires, Av. San Martín 4453, C1417DSE, Ciudad Autónoma de Buenos Aires, Argentina

## Abstract

The purpose of this study was to isolate and select indigenous soil *Pseudomonas* and *Bacillus* bacteria capable of developing multiple mechanisms of action related to the biocontrol of phytopathogenic fungi affecting soybean crops. The screening procedure consisted of antagonism tests against a panel of phytopathogenic fungi, taxonomic identification, detection by PCR of several genes related to antifungal activity, in vitro detection of the antifungal products, and root colonization assays. Two isolates, identified and designated as *Pseudomonas fluorescens* BNM296 and *Bacillus amyloliquefaciens* BNM340, were selected for further studies. These isolates protected plants against the damping-off caused by *Pythium ultimum* and were able to increase the seedling emergence rate after inoculation of soybean seeds with each bacterium. Also, the shoot nitrogen content was higher in plants when seeds were inoculated with BNM296. The polyphasic approach of this work allowed us to select two indigenous bacterial strains that promoted the early development of soybean plants.

## 1. Introduction

To increase crop yields, it is necessary to apply agrochemicals, which have several negative side effects [1]. Since pathogen damage potentially causes large yield losses, the use of plant growth-promoting bacteria (PGPB) with antifungal properties is an attractive alternative to the use of such xenobiotic compounds [2]. Species belonging to *Bacillus * and *Pseudomonas * are frequently used as biocontrol agents, since they excrete hydrolytic enzymes able to degrade cell walls [3], iron-chelating siderophores, and several cyclic lipodepsipeptides (LDP) [4].

*Pseudomonas * excretes a great variety of antibiotics such as 2,4-diacetylphloroglucinol (2,4-DAPG), pyoluteorin, pyrrolnitrin, and hydrogen cyanide [5, 6]. *Bacillus *strains also produce important antibiotics that are useful for plant disease control [7, 8]. Some of the well-documented PGPB characteristics related to soil fertility and plant nutrition optimization are the production of bacterial phytohormones and/or the solubilization of mineral phosphates [9, 10].

In Argentina, soybean (*Glycine max * [L.] Merr.) is the main grain crop, this country being the third soybean world's largest producer and the soybean flour and oil world's largest exporter. Diseases, pests, and weeds are the most important biotic factors that limit soybean yields in Argentina [11], and the main losses due to diseases are related to germplasm uniformity and lack of crop rotation [12].

The impact during the vegetative stages of development can have effects that will determine the health status of the crop during the reproductive stages [13]. Damping-off becomes relevant when sowing occurs in seasons characterized by cold, damp weather, since it results in stand reduction and contributes to increasing the weed control problem [14]. The strategies recommended to crop producers by the Instituto Nacional de Tecnología Agropecuaria (INTA) of Argentina are to avoid early sowing, introduce a rotation practice with gramineous crops, and use fungicide-impregnated seeds. Two major concerns associated with this last practice are the development of pathogen resistance and the secondary environmental impact produced by the presence of xenobiotic chemical compounds.

This study describes the isolation and selection of indigenous bacterial strains with antifungal activity from the soybean rhizosphere. Bacteria were identified and characterized based on their production of siderophores, biosurfactants, volatile compounds, auxins, hydrolytic enzymes, antibiotics, and phosphate solubilizing activity. We tested the protective effect provided by two strains on soybean plants challenged with *P. ultimum * when inoculated on seeds. Since soybean crops are being massively produced, this study may favourably contribute to the development of alternative and sustainable soybean production practices.

## 2. Materials and Methods

### 2.1. Sampling, Isolation, and Selection of Bacteria

Soil rhizosphere samples were obtained from soybean crops in the Province of Buenos Aires, Argentina. The primary screening for bacteria with antifungal activity was performed as follows: 1 g of rhizospheric soil sample was suspended in 5 mL of 0.9% (w/v) NaCl. After 1 minute of decantation, a loopful of the supernatant fraction was streaked on one edge of potato-dextrose-agar (PDA Merck) plates and incubated at 30°C. A 9 mm plug taken from the leading edge of *Fusarium oxysporum *BNM403 mycelium growing in PDA plates at 30°C was placed in the center of the plate 24 hours later. Plates were incubated at 30°C for six additional days. Then, bacteria were purified from plates where hyphal growth was inhibited [15]. Following this procedure, bacterial isolates were obtained and individually tested for hyphal growth inhibition. A cut-off of 40% hyphal inhibition was drawn, and the selected isolates were then examined for Gram reaction, endospore formation, and location within the cell (Gram+), or production of fluorescent pigments (Gram−) when grown in King's B (KB) agar plates [16].

### 2.2. Culture Conditions

Unless specified, all bacterial strains were grown in nutrient broth (NB, 3 g meat extract and 5 g peptone per liter) at 30°C for 24 hours, and shaking at 150 rpm. Before use, cultures were adjusted to 2 × 10^8^ CFU/mL. Bacteria were kept for long-term storage at −80°C in NB with 15% glycerol (v/v). The fungal strains were stored at 4°C in sterile soil. They were routinely grown on PDA at 24°C. Table 1 shows all the bacterial strains used in this paper.

### 2.3. Antagonism Against Pathogenic Fungi

Bacterial isolates were tested against *Macrophomina phaseolina, Sclerotinia minor*, *Fusarium oxysporum, Fusarium solani*, and *Pythium ultimum*.

### 2.4. Molecular Typing

Total genomic DNA was prepared from NB cultures by using the Wizard genomic DNA purification kit (Promega Inc., Madison, WI, USA) and adjusted to 50 ng/*μ*L. Isolates were characterized by rep-PCR genomic fingerprinting with BOXA1R primers [17]. *Pseudomonas *isolates were analyzed by PCR by using *Pseudomonas *sp. 16S rDNA specific primers, followed by multiple enzyme restriction fragment length polymorphism (MERFLP) [18]. For *Bacillus *isolates, 16S–23S rRNA IGS-PCR analysis was performed as previously described [8, 19]. Amplifications were performed in an MJ Research PTC-100 thermocycler using standard conditions. Total and partial sequencing of the 16S rRNA gene was performed by the Sequencing and Genotyping Service of the School of Exact and Natural Sciences, University of Buenos Aires, Argentina. The 16S rRNA gene sequences were compared with those in GenBank using the BLASTN 2.2.16 program [20], and strains that were closely related were aligned using the CLUSTAL W program [21].

### 2.5. Detection of Hydrolytic Enzymes

Chitinase activity was measured according to Chernin et al. [22], protease activity according to Berg et al. [23], and cellulolytic activity on microcrystalline cellulose-containing plates as described by Teather et al. [24].

### 2.6. Detection of Siderophore Production

Siderophore production was tested by growing *Pseudomonas *sp. and *Burkholderia cepacia *in the universal siderophore detection medium CAS agar [25].

### 2.7. Detection of Volatile Compounds

Briefly, *F. solani *BNM 400 was grown in either the presence or absence of bacteria with no physical contact between them, and mycelial growth was recorded, as described by Montealegre et al. [15]. The presence of the *hcnB *and *hcn*C genes encoding for hydrogen cyanide (HCN) synthase was evaluated by PCR. Hydrogen cyanide production was confirmed by assessing it with the paper disk method [26].

### 2.8. Detection of Pseudomonas Antibiotics

The presence of the following genes was evaluated by PCR as follows: *prn*C, related to the synthesis of pyrrolnitrin (PRN); *phl*D, involved in the synthesis of 2,4-diacetylphloroglucinol (DAPG); *phz*C and *phz*D, involved in the synthesis of phenazine-1-carboxylic acid (PCA); and *plt*C, related to pyoluteorin (PLT) biosynthesis. Thin layer chromatography (TLC) analysis following the protocols described previously was performed to detect pyrrolnitrin and pyoluteorin [27].

### 2.9. Screening of Biosurfactant Activity.

The presence of both *sfp*, which encodes for a 4′-phosphopantetheinyl transferase (PPTase) involved in the activation of surfactin synthase, and *asn,* which is related to mycosubtilin synthesis, were evaluated by PCR. The hemolytic activity of the cell-free supernatant was evaluated and 200 *μ*L samples were loaded into wells made in blood agar plates (bovine blood plates, Laboratorio Argentino, SA). After incubating for 48–72 h at 37°C, the hemolytic activity was evaluated as the size of clear halos around the wells [7]. TLC was performed on silica gel plates to confirm the presence of biosurfactant and other lipopeptides of *Bacillus*-like isolates, as previously described by Souto et al. [8].

### 2.10. PCR Reaction Conditions

DNA purification was performed as mentioned above. PCR reactions were performed in a 25 *μ*L reaction mixture containing 20 ng DNA, 200 *μ*mol/L of each dNTP (Invitrogen Co.), 1.5 or 2.5 mmol/l MgCl2 (Promega, Inc.) depending on the pair of primers used, 0.2 *μ*mol/L of each primer, 1U Taq DNA Polymerase (Invitrogen Co.), and the buffer provided with the enzyme. The primers and annealing temperature used in these experiments were as follows: prnC-F and prnC-R, 67°C [28]; phl2-F and phl2-R, 61°C; pca-F and pca-R, 61°C [5]; pltc-F and pltc-R, 61°C [27]; ACa and ACb, 68°C [6]; sfp-F and sfp-R, 53°C [29]; and ASnI-F and ASnI-R, 55°C [30]. PCR products were separated on 1% agarose gel in Tris-Borate-EDTA buffer (89 mmol/L Tris, 89 mmol/L boric acid, 2 mmol/L EDTA, and pH 8.0) at 5 V/cm for 2 h.

### 2.11. Measurement of Auxin Production

Auxin production was determined by the colorimetric method described by Kamilova et al. [31] with some modifications. The isolates were grown in M9/glycerol medium at 30°C, either with or without tryptophan (100 *μ*g/mL). After 1, 5, and 8 days, the amount of auxins in bacterial cultures was determined using the Salkowski reagent [32] and indole-3-acetic acid (IAA, Sigma-Aldrich Corp.) as standard.

### 2.12. Detection of the Phosphate Solubilizing Activity

Phosphate solubilizing activity was assessed on yeast extract dextrose-CaHPO4 agar plates by measuring the clear zone surrounding the developed bacterial colony, after 7 days of incubation at 30°C [9].

### 2.13. Colonization Assay

This experiment was performed under aseptic conditions. Soybean seeds were surface sterilized as described by Cattelan et al. [33] and left for 1 h either in the water of the last wash or in contact with a bacterial suspension. Treated seeds were introduced into sterile tubes with Hoagland's nutrient solution. Seedlings were kept in a growth chamber at 25°C with a photoperiod of 16 h/8 h (light/dark). Six replicate tubes were used for each treatment. After seven days, plant roots were separated from the shoots, washed with 5 mL of sterile water, immersed into 5 mL of sterile water, vigorously shaken for 30 second, and sonicated in an ultrasonic bath (Grant Instruments Ltd, Cambridge, UK) for 3 min, in order to quantify the loosely attached bacteria, by colony count on agar plates. The roots were washed again and suspended in 3 mL of sterile water, dispersed with a hand mortar to quantify strongly attached bacteria, and then, roots were dried and weighed.

### 2.14. Microcosm Assays

Three selected strains, *P. fluorescens *BNM296, BNM297, and *B. amyloliquefaciens *BNM340, were tested as seed inoculant components. Controls consisted of noninoculated seeds. Treatments consisted of seeds inoculated with BNM296, BNM297, or BNM340 and either infested or not with *P. ultimum *of the nonsterile agricultural soil used in 0.9 L-pots. Six pots per treatment were used in a completely randomized design. An infested substrate was prepared by inoculating sterile vermiculite with mycelia of *P. ultimum *(5 × 10^4^ propagules/g). Ten soybean inoculated seeds (5 × 10^5^ CFU/seed) were sown per pot. Then, 10 g of fungal inoculum was added as a layer and another layer of sterile vermiculite was spread over the already added rooting medium in each pot. Seeds were left in contact for 1 h with each culture, dried at room temperature, and sown in the pots. Plants were harvested after 20 days of growth with a photoperiod of 16 h/8 h (light/dark) at 22°C. Roots of all plants were washed and severity of root rot was determined visually using a rating scale of 0–4 [34], where 0 = no disease symptoms, 1 = 1%–25% (growth retardation), 2 = 26%–50% (moderate damping-off), 3 = 51%–75% (severe damping-off), and 4 = 76%–100% (pregermination seed decay). The disease severity index was used to calculate the percentage suppression of root, using the following equation: % suppression = [(*A* − *B*)/*A*] × 100, where *A* = disease severity exhibited in the root region due to *P. ultimum *alone and *B* = disease severity exhibited in the root region after inoculation with both the pathogen and bacterial antagonists. Also, shoot dry weight and total nitrogen content of shoots were determined as described by Smith [35].

### 2.15. Nucleotide Sequence Accession Numbers

The Gene Bank database accession numbers of the partial 16S rDNA sequences determined are as follows: DQ885200 for *Burkholderia cepacia *BNM345, DQ885199 for *B. cepacia *BNM353, EF095217 for *B. cepacia *BNM299, EF095218 for *Bacillus cereus *BNM343, and FJ513627 *Pseudomonas fluorescens *BNM296.

### 2.16. Data Analysis

Data were analyzed with the Info Stat professional Version 1.1 software from the Universidad de Córdoba, Estadística y Diseño FCA, Argentina, before statistical analysis logit and square root transformation were applied to emergence rate and colonization data, respectively, to normalize the error variances; when required, the ANOVA protected least significance difference (LSD) test and specific contrasts were used. Differences were considered significant when *P *< 0 .1 or *P *< 0 .05.

## 3. Results

### 3.1. Isolation and Identification

A primary selection was made from the antagonism test plates where the confluent growth of bacteria from the soybean rhizosphere inhibited the development of fungal mycelia. Pure bacterial cultures isolated from those plates were tested for fungal antagonism. This procedure resulted in 150 initial isolates that inhibited *Fusarium solani *BNM400 more than 40% with respect to the fungi growing alone. By applying additional selective criteria as described in Materials and Methods, the number of selected isolates was reduced to 80. Looking for bacteria with a wide range of antifungal action, six out of the 80 isolates were reselected after testing them against a panel of phytopathogenic fungi (Table 2). These six strains were subjected to molecular typing. An IGS-PCR fingerprint analysis allowed the identification of several species of the *Bacillus *group. The BNM340 isolate had a pattern identical to those of the reference strains of *B. amyloliquefaciens *DSM1060, DSM7^T^, and BNM122. IGS-PCR, rep-PCR, and 16S rDNA sequence analyses of BNM343 strain showed the same pattern as that shown by the members of the *B. cereus *group [36]. In the case of *Pseudomonas*-like bacteria, only BNM296 and BNM297 strains amplified the expected 990-bp fragment of the 16S rRNA gene and can therefore be classified as *Pseudomonas *sp. Their MERFLP patterns were identical to each other but not to other *Pseudomonas *used for comparison, as previously described [18]. The result of the biochemical test was similar to those displayed by *Pseudomonas fluorescens *species [37]. Other isolates, such as BNM345 and BNM299, were classified as belonging to *Burkholderia cepacia *by means of the 16S rRNA gene sequence similarity [38]. These two isolates shared 99% sequence similarity with the reference strains *Burkholderia cepacia *AMMD and *Burkholderia cenocepacia *HI2424.

### 3.2. Isolate Characterization

Table 3 summarizes the results of the characterization of the six selected strains. *Pseudomonas *and *Burkholderia* produced siderophores. Regarding the cell wall degrading activity, Table 3  shows the widespread distribution of chitinolytic activity in the different strains. All isolates had low cellullase activity, with the exception of *P. fluorescens *strains, which had none. High proteolysis was distributed almost equally among all isolates. Production of volatile compounds was detected in BNM296 and BNM297 as well as in *B. cepacia* strains BNM299 and BNM345. The presence of *hcn*BC genes was evidenced by PCR for BNM297 but not for BNM296. This result was consistent with the paper disk test for cyanide production (data not shown), thus indicating that the synthesis of HCN occurred in BNM297 but not in BNM296. With respect to antibiotic production, a positive signal for *prn*C was detected in *P. fluorescens *BNM296, and *B. cepacia *BNM299 and BNM345. The search for the presence of genes related to the synthesis of DAPG and PCA resulted in no positive signals for any strain. A positive signal for *plt*C was detected in *P. fluorescens *BNM296 and *B. cepacia *BNM299. The ability to produce PRN and PLT was confirmed in each case by TLC analysis. Antibiotics were identified by their characteristic colors and Rf values, corresponding to those of standards PLT, Rf: 0.65 cm, brown and PRN, Rf: 0.81 cm, and pink (data not shown). When grown in blood agar plates, all strains except BNM345 showed a hemolytic zone. The PCR screening for sequences related to *Bacillus *lipopeptide synthesis showed the presence of the *asn *and *sfp *genes in *B. amyloliquefaciens *BNM340. Several spots of amphipathic compounds, two of which corresponded to surfactin and iturin A, were detected by TLC analysis; the others have not yet been identified. Auxin production measured as IAA equivalents was detected for all isolates. The phosphate solubilizing activities detected were considered as relevant for BNM296 and BNM299 whereas no or very low activity was present in the remaining bacteria.

### 3.3. Soybean Seed and Root Inoculation Assays

*B. amyloliquefaciens *BNM340 and *P. fluorescens *BNM296 and BNM297 were selected to carry out inoculation assays. Despite their high antagonistic effectiveness,* B. cepacia *and *B. cereus *isolates were not included in any further experiments, because of their genetic relationship with potentially hazardous bacteria [39, 40].

Results on soybean seed and root colonization are presented in Table 4. The quantification of bacteria adhering to the surface of inoculated seeds resulted in a concentration of BNM296 or BNM297 higher than that of BNM340. 

Regarding the two types of colonizing modes of the roots mentioned here, we determined that in the case of BNM340, both the strongly and loosely bound bacterial fractions were significantly lower than those for BNM296 and BNM297. For BNM340, BNM296 and BNM297, there were no significant differences between CFU/mg dwr recovered of strongly and loosely attached fractions. No seed-borne bacterial colonies were recovered from noninoculated seeds or seedling controls.

### 3.4. Microcosm Assays

When plants were challenged with the pathogen, *B. amyloliquefaciens *BNM340 was the most effective strain suppressing damping-off, followed by *P. fluorescens *BNM296 (Table 5). *P. fluorescens *BNM297 was unable to exert any effect on soybean plants, which reacted like control plants growing in soils containing only the fungi.

Plants inoculated with BNM296 developed better when compared to the control plants in the non-infested soil. The emergence rate of plants in the infested soil inoculated with either BNM340 or BNM296 showed the same rate as the plants in the non-infested soil. The failure of BNM297 to protect plants was also evidenced by this parameter. Plants biomass was higher for BNM296 in the non-infested soil than for the other treatments; and although there was a tendency to have a higher biomass for the other inoculation treatments as compared to controls, it was not statistically significant. When plants were challenged with *P. ultimum*, the plants weights recorded were similar to those in the non-infested soil, except for BNM297. The nitrogen content of plants inoculated with BNM296 was higher than that of the rest of treatments, in both situations. No differences were detected between the other treatments. 

## 4. Discussion

The screening strategy carried out in this paper consisted of the isolation of culturable bacteria strains capable of stimulating plant growth through biocontrol mechanisms. Accepting what was earlier stated by Whipps [1, 41] and other authors [2] that effective biocontrol agents often act through the combination of several different mechanisms, a selection procedure that allowed us to find strains that were positive for more than one antagonistic mechanism was designed. The primary screening resulted in a group of bacteria able to survive in the presence of other microorganisms and display a nonobligate bacterial predator behavior [42], thus allowing us to select bacteria showing several antagonistic mechanisms.

We focused on bacterial genera that are often found in large populations in soils with general disease suppression [43], such as positive spore-forming Gram-positive species belonging to the *Bacillus *genus and Gram-negative ones belonging to *Pseudomonas. *In this context, the most relevant isolates belonged to *P. fluorescens*, *Burkholderia cepacia *group, *B. amyloliquefaciens*, and *B. cereus*. Our results are consistent with the early raised hypothesis that this group of microorganisms is responsible for this kind of phenomenon in the soil. In addition, this is supported by the reports by Adesina et al. [44] and Kuklinsky-Sobra et al. [45], which have focused on the soybean-associated soil of the South American region.

Although *B. cereus *and *B. cepacia *isolates displayed interesting phenotypic characteristics as potential PGPB, we decided not to further analyze their behavior in seed inoculation assays since the differentiation between agricultural biopesticides and pathogenic species is still unclear [39, 40]. We therefore characterized three strains as follows: two *P. fluorescens*, BNM296 and BNM297, and one *B. amyloliquefaciens *BNM340.

Cell-wall degrading activities seemed to be the mechanisms responsible for *B. amyloliquefaciens *BNM340 antagonism, since several enzymatic activities (proteolytic, chitinolytic and cellulolytic) were detected and also because this strain excreted surfactin and some iturin-like lipodepsipeptides, such as iturin A. These mechanisms have been previously correlated with antifungal activity [4, 46]. As previously described for *Pseudomonas *[43], *P. fluorescens *antagonism is related to mechanisms more diverse than those found for *Bacillus. *The traits found included siderophores, volatile compounds, antibiotics such as PRN and PLT, cell-wall degrading molecules, extracellular chitinase, and protease enzymes, all proved to be involved in antagonistic activities [4, 27, 28]. The differences found between these strains were the production of HCN by BNM297 and not by BNM296 and the production of PRN and PLT by BNM296 but not by BNM297. We measured the enhancement of early soybean development trying to correlate these results to the most significant bacterial properties [33, 45]. Although BNM296, in addition to biocontrol properties, was able to dissolve phosphate, it is impossible to correlate this result with plant growth enhancement. The halos produced by BNM296 during the assay were comparable to those of the PGPB strains analyzed by de Freitas et al. [9] on rhizosphere bacteria from canola and in soybean rhizosphere by *Bradyrhizobium *strains studied by Fernández et al. [47]. Since the three strains were able to produce auxins in the conditions tested and although no direct indication that plant growth enhancement was due to their excretion, it is more likely that this phenomenon was due to the presence of these compounds. In addition, we should consider that the amounts of auxins detected were similar to those previously described for PGPB by Kang et al. for *P. chlororaphis *[48] and by Idris et al. for *B. amyloliquefaciens *[49].

The results of the colonization of the soybean rhizosphere confirm reports on the interactions of *B. amyloliquefaciens *and *Pseudomonas *strains, supporting their use as rhizosphere colonizers [48, 50, 51].

*B. amyloliquefaciens *BNM340-inoculated soybean plants were protected from a high *P. ultimum *infestation, since only 30% of seedlings emerged in the control treatments. The storage and persistence of *Bacillus *in the soil make this strain a very good candidate to be included in inoculant formulations.

Our results point to *P. fluorescens *BNM296 as a soybean early growth promoter. Inoculation resulted in healthier and larger plants. The increase in nitrogen content was significant and there are not many reports about antifungal strains sharing this characteristic [46, 52]. This increase in nitrogen content could be attributed only to the increased capacity of the plants to incorporate fixed nitrogen.

The fact that strain BNM297 was unable to protect plants against damping-off correlated well with the antagonism assay since it had a weak response against *P. ultimum *in Petri dishes. The differences in antibiotic production between BNM296 and BNM297 may explain why plants responded differently to the inoculation and further validate the screening procedure designed. Nonetheless, the selected strains must be subjected to further analysis, such as inoculation of plants challenged with other pathogens.

The screening procedure demonstrated to be very effective. Although we did not establish a direct relationship between the described mechanisms and the protection that these strains demonstrate towards damping-off, the main goal of this work was accomplished and it shows a promising beginning for the formulation of inoculants that include indigenous bacteria. The practical significance of this type of studies acquires its real importance when considering the need to replace fumigants and other chemical control procedures for the treatment of soil and/or plant diseases.

## Figures and Tables

**Table 1:** Bacterial species source and origin.

Strain	Source
*Azospirullum brasilense *Sp7 (ATTC 29145)	ATCC^§^
*Bacillus amyloliquefaciens* BNM*122	Sunflower capitulum [8]
*B. amyloliquefaciens* DSM1060	DSMZ^†^
*B. amyloliquefaciens* DSM7^T^	DSMZ
*Bacillus licheniformis* DSM1913	DSMZ
*Bacillus megaterium* DSM337	DSMZ
*Bacillus subtilis subtilis* DSM10	DSMZ
*Bacillus sp. *DSM 1325	DSMZ
*Bradyrhizobium japonicum* USDA138	NRRL^‡^
*Pseudomonas aeruginosa* ATCC27587	ATCC
*Pseudomonas fluorescens* ATCC17397	ATCC
*Pseudomonas fluorescens* DSM50090^T^	DSMZ
*Pseudomonas putida *KT2440	ATCC
*Bacillus amyloliquefaciens* BNM340	This study, agricultural soil
*Bacillus cereus* BNM343	This study, agricultural soil
*Burkholderia cepacia* BNM299	This study, agricultural soil
*B. cepacia* BNM345	This study, agricultural soil
*Pseudomonas fluorescens* BNM296	This study, agricultural soil
*P. fluorescens* BNM297	This study, agricultural soil

*Banco Nacional de Microorganismos, INBA-CONICET, Argentina, ^†^German Resource Centre of Biological Material, Germany; ^‡^Agricultural Research Service Culture Collection, USA; ^§^American Type Culture Collection, USA.

**Table 2:** Antagonism of isolates against different pathogenic fungi.

		BNM	BNM	BNM	BNM	BNM	BNM
		340	343	297	296	299	345
Ascomycota	*M. phaseolina* BNM401	+++	+++	+++	−	−	+++
	*S. minor *BNM402	++	++	+	++	++	++

Mitosporic Fungi	*F. oxysporum* BNM403	++	++	++	++	++	++
	*F. solani* BNM400	++	++	++	+++	++	++
	*F. solani *BNM405	++	++	++	−	+	++
	*F. solani *BNM406	++	++	++	+	−	++
	*F. oxysporum *BNM404	++	++++	++	+	++	++

Oomycota	*P. ultimum* BNM407	+++	++	+	++	++	++

Relative growth in the presence or absence of bacteria as follows: same as control, (−); 99% to 61%, (+); 60% to 31%, (++); 30% to 1%, (+++); total inhibition, (++++). Experiments were repeated three times for each bacterial fungal combination on PDA medium. Bacterial strains used: *B. amyloliquefaciens* BNM340, *B. cereus* BNM343, *P. fluorescens* BNM296 and BNM297, and *B. cepacia *BNM299 and BNM345. Siderophore producers (BNM296, 297, 299, and 345) were also tested in KB medium and were found to be effective antagonists in comparison with the results in PDA. The fungal strains tested also belonged to the Banco Nacional de Microorganismos, INBA-CONICET, Argentina (BNM).

**Table 3:** Main characteristics of the selected strains.

Characteristic	BNM340	BNM343	BNM297	BNM296	BNM299	BNM345
Siderophores*	nd	nd	+	+	+	+
Chitinolytic^†^	4.5 ± 0.7	9.0 ± 1.4	6.8 ± 1.1	2.5 ± 0.7	3.5 ± 0.7	4.0 ± 0.0
Cellulolytic^‡^	5.0 ± 1.2	4.2 ± 0.3	0	0	5.8 ± 0.5	5.3 ± 0.5
Proteolytic^§^	31.0 ± 1.4	31.5 ± 2.1	31.5 ± 2.1	35.0 ± 5.7	29.5 ± 2.1	35.5 ± 0.7
Volatile comp.**	nd	nd	0.6 ± 0.2	0.6 ± 0.2	1 ± 0.1	0.7 ± 0.2
Antibiotics^††^	nd	nd	−	+	+	+
Biosurfactant^‡‡^	+	++	+/−	+/−	+	−
Auxins^§§^	8.4 ± 4.1	11.3 ± 6.0	4.12 ± 0.2	3.4 ± 0.8	1.0 ± 0.3	1.2 ± 0.3
P solubilization***	−	−	−	+	+	+/−

Results of the first screening tests for characterization of *B. amyloliquefaciens* BNM340, *B. cereus* BNM343, *Pseudomonas sp*. BNM296, and BNM297, *B. cepacia* BNM299 and BNM345.*+, Siderophore detected after 72 h at 30°C, + orange halos between 1–5 mm.^†^Mean halo diameter (mm) and SE of chitin consumption on agar-chitin plates. ^‡^Mean halo diameter (mm) and SE of cellulose consumption on microcrystalline cellulose plates. ^§^Mean halo diameter (mm) and SE of casein consumption on skim milk plates. **Relative growth of* Fusarium solani* in the presence of isolates but with no physical contact with them. ^†† ^PCR analysis of different compounds related to antibiosis. ^‡‡^hemolytic halo, +/− halo radius between 0–1 mm, + halos between 1–5 mm, ++ halos >5mm on blood-agar plates. ^§§^Mean and SD amount of IAA (*μ*g/mL) in the spent culture medium after eight days of incubation of the three independent experiments performed. ***Solubilization of CaHPO4, +: solubilization halo ≥10 mm, +/− : halo <10 mm, − : no solubilization halo, nd: not determined.

**Table 4:** Seed and root colonization of isolates selected to perform microcosm assays.

	Seed colonization	Root colonization	
	(log CFU/seed)	Loosely attached	Strongly attached
		(log CFU/mg dwr)	(log CFU/mg dwr)
BNM296	6.65 ± 0.09^a^	6.85 ± 0.33^a^	6.9 ± 0.66^a^
BNM297	6.63 ± 0.17^a^	6.73 ± 0.14^a^	6.65 ± 0.16^a^
BNM340	5.73 ± 0.66^b^	5.18 ± 1.20^b^	4.74 ± 0.33^b^

Mean ± SE values of three independent experiments. Means within columns followed by the same letters do not differ significantly, (*P* < 0.05) according to Tukey's multiple comparison test using the Infostat software.

**Table 5:** Inoculation of soybean seeds with the potential biocontrol agents, response of plant growth in soil infested with *Pythium ultimum*.

Treatment	% Disease suppression*	Emergence rate (%)^†^	Shoot dry wt. (g/plant)^‡^	% Nitrogen/plant^§^
Control	85.6 ± 19.4^cd^	93 ± 01^b^	2.31 ± 0.30^a^	1.72 ± 0.17^a^
BNM296	95.88 ± 10.1^d^	97 ± 03^b^	2.72 ± 0.43^b^	2.62 ± 0.19^b^
BNM297	86.6 ± 12.6^cd^	90 ± 01^b^	2.39 ± 0.28^ab^	1.70 ± 0.25^a^
BNM340	86.6 ± 12.6^cd^	93 ± 01^b^	2.38 ± 0.32^ab^	1.74 ± 0.16^a^
P	0.20 ± 16.7^a^	31 ± 11^a^	1.53 ± 0.19^c^	1.66 ± 0.20^a^
P+BNM296	53.7 ± 26.4^b^	90 ± 04^b^	2.28 ± 0.42^ab^	2.49 ± 0.21^b^
P+BNM297	1.20 ± 21.4^a^	33 ± 16^a^	0.60 ± 0.73^d^	1.68 ± 0.13^a^
P+BNM340	74.07 ± 22.0^c^	89 ± 06^b^	2.54 ± 0.22^ab^	1.85 ± 0.13^a^

*Percent disease suppression was calculated using [(*A* − *B*)/*A*] × 100, where *A* = root rot severity index exhibited by the control treatment inoculated with the pathogen alone and *B* = root rot severity index exhibited by plants treated with both the pathogen and the bacterial antagonists. Control = non-inoculated seeds in non-infested substrate; *P* = substrate infested with *P. ultimum*; BNM296, BNM297, and BNM340; seeds inoculated with *P. fluorescens *BNM296, *P. fluorescens *BNM297 or* B. amyloliquefaciens *BNM340 in non-infested substrate; and P+BNM296, P+BNM297 and P+BNM340, seeds inoculated with BNM296, BNM297, or BNM340 in substrate infested with *P. ultimum. *Means within columns followed by the same letters do not differ signi*ﬁ*cantly (*P* < 0.10, MSD = 17.9). ^†^Means ± SE (*P* < 0 .05, MSD = 0.2). ^‡^Means ± SE (*P* < 0 .05, MSD = 0.73). ^§^Means ± SE (*P* < 0 .1, MSD = 0.44) all according to the LSD test using the Infostat software.
